# Heart failure in erythrodermic psoriasis: a retrospective study of 225 patients

**DOI:** 10.3389/fcvm.2023.1169474

**Published:** 2023-08-01

**Authors:** Chao Wu, Chenyang Yu, Yuyan Yang, Hongzhong Jin

**Affiliations:** Department of Dermatology, State Key Laboratory of Complex Severe and Rare Diseases, Peking Union Medical College Hospital, Chinese Academy of Medical Sciences and Peking Union Medical College, National Clinical Research Center for Dermatologic and Immunologic Diseases, Beijing, China

**Keywords:** erythrodermic psoriasis, erythroderma, psoriasis, heart failure, fibroblast growth factor 23 (FGF23)

## Abstract

**Purpose:**

Erythrodermic psoriasis (EP) is a severe form of psoriasis that affects multiple organs, including the cardiovascular system. However, few studies have focused on this condition.This study is aimed to assess the prevalence and factors associated with heart failure in EP patient, and to the measure the serum concentrations of fibroblast growth factor 23 (FGF23), a potential predictor of chronic heart failure.

**Methods:**

We retrospectively studied patients with EP hospitalized at Peking Union Medical College Hospital between January 2005 to October 2021. The prevalence of heart failure and associated factors was measured. In addition, peripheral blood samples were collected from 17 patients and matched with samples from eight healthy controls, and their serum concentrations of FGF23 were measured by ELISA.

**Results:**

We studied 225 patients with EP, with a male: female ratio of 2.7:1 and a mean age of 47.6 ± 16.7 years. Twenty-five (11.1%) participants were diagnosed with heart failure during their hospital stay. The patients with EP and heart failure were older (58.2 years vs. 46.2 years, *p *= 0.001); had a higher prevalence of a history of coronary heart disease (32.0% vs. 21.5%, *p *< 0.001), fever (48.0% vs. 23.0%, *p *= 0.007), infection (56.0% vs. 35.5%, *p *= 0.046); higher hsCRP concentration (43.2 mg/L vs. 8.2 mg/L, *p *= 0.005); and higher prevalence of anemia (60.0% vs. 22.0%, *p *< 0.001), hypoalbuminemia (64.0% vs. 42.0%, *p *= 0.037), and hyperlipidemia (40.0% vs. 20.0%, *p *= 0.023) than those without heart failure. The serum FGF23 concentration was significantly higher in patients with EP than controls (493.1 pg/ml vs. 277.8 pg/ml, *p *= 0.027), and was significantly lower after treatment (395.7 pg/ml vs. 463.1 pg/ml, *p *= 0.022).

**Conclusions:**

Clinicians should be aware of the risk of heart failure in patients with EP, and especially those of advanced age and with a history of coronary heart disease, severe systemic symptoms, high concentrations of inflammatory biomarkers, and poor nutritional status.

## Introduction

Erythrodermic psoriasis (EP) is one of the most severe and rare subtypes of psoriasis, accounting for approximately 1%–2% of cases of psoriasis (1–3). The prevalence of EP is higher in Asian than Caucasian populations (4). Patients with EP present with erythematous, scaly cutaneous lesions covering >90% of their body surface area (5). Owing to the extensive disruption of the skin barrier, patients with EP often have symptoms reflecting multi-organ, systemic involvement, such as dehydration, electrolyte disturbances, fever or hypothermia; and in severe cases, high output heart failure, pulmonary edema, and severe infections, which can be life-threatening (1, 5–7). The mortality rate has been reported to range from 9% to 64% (7).

An additional 6.2% absolute risk of a cardiovascular event over a 10-year period was demonstrated in patients with severe psoriasis, when compared to the general population (8). A systematic analysis of 2,941 patients with psoriasis showed that they have a significantly higher prevalence of heart failure than patients with other skin diseases (9). Furthermore, the prevalence of left ventricular dysfunction was found to be significantly higher in patients with psoriasis than in healthy controls (10, 11). One previous cohort study showed that the 10-year risk of major adverse cardiovascular events (MACE) attributable to severe psoriasis is approximately 6% (8). Another population study performed in Denmark showed that psoriasis is associated with a higher risk of MACE, similar to that associated with the conventional cardiovascular disease risk factor diabetes mellitus (12). In addition, patients with EP have been reported to be at risk of high-output heart failure (13, 14). However, owing to the low incidence of EP, few large studies have focused on its association with cardiovascular disease.

Fibroblast growth factor 23 (FGF23) is a member of the fibroblast growth factor family that has a molecular weight of 32 kDa and plays an endocrine role in the regulation of phosphate and vitamin D metabolism. Several previous studies have shown that the circulating FGF23 concentration is not only associated with cardiac complications in patients with chronic kidney disease but is also independently associated with cardiac disease (15). In humans, high circulating FGF23 concentrations have been shown to be significantly associated with left ventricular hypertrophy (16). In addition, FGF23 was shown to directly induce pathological hypertrophy in isolated cardiomyocytes (17). Finally, high FGF23 has been shown to be an accurate predictor of chronic heart failure events (18, 19) and fatal outcomes in patients with heart failure and reduced ejection fraction (HFrEF) (20). However, few studies have investigated serum FGF23 concentrations in patients with psoriasis. Gökhan et al. measured the serum FGF23 concentrations of 45 patients with plaque psoriasis and reported a mean concentration of 272.8 pg/ml, which was significantly higher than that of healthy controls (*p *< 0.001) (21). However, no correlation was found between the FGF23 concentration and the psoriasis area and severity index (PASI). No studies have investigated FGF23 in patients with EP.

In summary, although the association between psoriasis and cardiovascular disease is now widely accepted, few studies have focused on heart failure in the most severe subtype of psoriasis, EP. Therefore, in the present study, we aimed to assess the prevalence of and factors associated with heart failure in patients with EP, and to measure their serum FGF23 concentrations, to determine whether this might represent a useful predictor of chronic heart failure events in patients with EP.

## Materials and methods

### Study sample

We performed a retrospective case-control study using clinical data collected for patients with EP who were hospitalized in the Department of Dermatology at Peking Union Medical College Hospital between January 2005 and October 2021. The inclusion criteria were as follows: (i) diffuse erythema, with or without scaling, covering >90% of the body surface area; and (ii) a clear history of psoriasis, skin lesions typical of psoriasis, or skin histopathological findings of classical psoriasis-like changes. Patients enrolled should meet both two criteria. The exclusion criteria were as follows: (i) a concurrent diagnosis or history of another cause of erythroderma; (ii) admission to another ward, with incomplete clinical information; (iii) transfer to another ward or sudden discharge requested by the patient; and (iv) patients who had a history of heart failure before hospitalization. The study protocol was approved by the ethics committee of PUMCH (approval number S-K1526). Written informed consent was obtained from each patient in our study. All procedures in this research followed the ethical criteria of the responsible committee on human experimentation (institutional and national), as well as the Helsinki Declaration of 1975, as amended in 2000.

### Data collection

Anonymized information regarding the demographic features, clinical manifestations, physical examination results, and laboratory and imaging results were retrospectively collected from the patients' medical records. The demographic and clinical features collected were age; sex; body mass; height; heart rate; blood pressure; history of smoking and alcohol consumption; duration of EP; and the presence or absence of fever, edema of the lower limbs, superficial lymphadenopathy, pruritus, swelling, exudation, and symptoms related to the cardiovascular system (palpitations, chest tightness, breathlessness, and oliguria). The laboratory data collected were blood cell counts, biochemical data (liver enzyme activities, indices of renal function, and serum lipid concentrations), erythrocyte sedimentation rate (ESR), high-sensitivity C-reactive protein (hsCRP) concentration, myocardial enzyme activities (creatine kinase, creatine kinase MB-mass, cardiac troponin I, and N terminal pro B type natriuretic peptide), and the concentrations of pro-inflammatory cytokines (IL-6, IL-8, IL-10, and TNF-α). Electrocardiographic and echocardiographic data were also collected. Body mass index (BMI), mean arterial pressure (MAP), and the estimated glomerular filtration rate (eGFR) were calculated.

Heart failure was diagnosed based on the Framingham criteria (22, 23). Additionally, all patients diagnosed with heart failure also had supporting evidence of echocardiography and/or plasma NT-proBNP levels. In details, the echocardiographic findings indicated ventricular systolic or diastolic insufficiency. The plasma NT-proBNP concentration exceeded the appropriate cut-off value for the age group (450 pg/ml for patients <50 years old, 900 pg/ml for patients 50–75 years old, and 1,800 pg/ml for patients >75 years old) (24). The diagnosis of heart failure was made mainly by internal consultant, and by dermatologists in a few cases.

The disease severity was evaluated according to the criteria designed specifically for EP (25). Patients were defined as having moderate-to-severe EP if they had at least two of the three following characteristics, and as having mild EP if they had fewer than two characteristics: (i) body temperature >37.3°C upon admission; (ii) swelling and exudation was a feature of >50% of the skin lesions or lower extremity oedema; and (iii) superficial lymphadenopathy.

The diagnosis of infection was made based on the clinical manifestations, the results of laboratory tests and/or imaging examinations.

### Serum collection and measurement of FGF23 concentration

Serum samples were collected from 17 patients with EP who visited the outpatient department of PUMCH between 2019 and 2020 and who fulfilled the inclusion criteria listed above. Serum samples were collected from all 17 participants before treatment and from five patients after treatment, when ≥80% of the skin lesions had resolved. After an overnight fast of ≥8 h, venous blood samples were obtained, allowed to clot, and centrifuged at 2,000 × g for 10 min to obtain serum. The FGF23 concentration of each was measured using a commercially available ELISA kit (Ruixin Biological Technology Co., Shanghai, China).

### Statistical analysis

Continuous data are summarized as the mean (standard deviation, SD) when normally distributed and as the median (interquartile range, IQR) when not normally distributed. Categorical data are displayed as a number and proportion. Comparisons between two groups were made using unpaired *t*-tests when the data were normally distributed and the Mann–Whitney *U*-test when they were not. Categorical data were analyzed using the *χ*^2^ test or Fisher's exact test and are reported as relative risks with 95% confidence intervals (CIs) and *p* values. The relationships of demographic characteristics, clinical manifestations, and laboratory data with the serum concentration of FGF23 were assessed using Pearson's or Spearman's correlation analysis. To exclude confounding factors, the binary logistic regression was performed, incorporating the variables with significant differences in comparative analysis, using Forward (LR) for variable screening. All the tests were two-tailed, and *p* < 0.05 was considered to represent statistical significance. We used SPSS version 15.0 software (SPSS, Chicago, IL, USA) for the statistical analyses.

## Results

### Characteristics of the participants

We studied a total of 225 participants, with a male-to-female ratio of 2.7:1 and a mean age of 47.6 years at admission. Of these, 10 (4.4%) had primary EP and the remaining 215 (95.6%) had a positive history of other types of psoriasis. Sixty-seven participants (29.8%) were admitted because of relapse. The mean age of onset of psoriasis was 33.1 ± 16.7 years, and the mean duration of psoriasis was 14.5 ± 11.4 years. The median duration of EP was 4 weeks. Regarding their clinical manifestations, 46 of the participants had a mean PASI score of 42.3 ± 12.3 and 79 (35.1%) were categorized as having moderate-to-severe psoriasis. A complete list of the characteristics of the participants is provided in Table 1.

**Table 1:** Demographic and clinical features of 225 EP patients.

Characteristics	*n* = 225
Gender	
Male (*n*, %)	164 (72.9%)
Female (*n*, %)	61 (27.1%)
Age at admission (years) (average ± SD)	47.6 ± 16.7
Length of stay (days) (average ± SD)	25.3 ± 14.5
BMI (kg/m^2^)	24.8 ± 4.2
Sources of EP (*n*, %)	
Primary EP	10 (4.4%)
Transformed from other types of psoriasis	215 (95.6%)
Age of psoriasis onset (years) (average ± SD)	33.1 ± 16.7
Duration of this episode of EP (weeks) [median, (Q1, Q3)]	4.0 (3.0, 12.0)
Primary lesion (*n*, %)	
Trunk and limbs	144 (65.2%)
Limbs	37 (16.7%)
Trunk	20 (9.0%)
Head	7 (3.2%)
Trunk, limbs, and head	6 (2.7%)
Limbs and head	3 (1.4%)
Trunk and head	3 (1.4%)
Genitalia	1 (0.5%)
Clinical manifestations	
PASI score (average ± SD)	42.3 ± 12.3
Fever (*n*, %)	58 (25.8%)
Lower limbs edema (*n*, %)	129 (57.3%)
Chest tightness (*n*, %)	9 (4.0%)
Palpitation (*n*, %)	10 (4.4%)
Precordial distress (*n*, %)	2 (0.9%)
Oliguria (*n*, %)	3 (1.3%)
Disease severity^a^	
Mild (*n*, %)	146 (64.9%)
Moderate-to-severe (*n*, %)	79 (35.1%)
Laboratory examinations	
WBC count (×10^9^/L) (average ± SD)	8.48 ± 3.17
NEUT count (×10^9^/L) (average ± SD)	5.67 ± 2.80
Hgb (g/L) (average ± SD)	126.2 ± 19.9
Alb (g/L) (average ± SD)	34.9 ± 5.7
LDH (U/L) (average ± SD)	204.7 ± 77.5
eGFR (ml/min/1.73 m^2^) (average ± SD)	110.2 ± 32.3
TC (mmol/L) (average ± SD)	3.89 ± 1.00
TG (mmol/L) (average ± SD)	1.45 ± 0.81
HDL-C (mmol/L) (average ± SD)	0.90 ± 0.24
LDL-C (mmol/L) (average ± SD)	2.41 ± 0.80
ESR (mm/h) [median, (Q1, Q3)]	16.50 (8.00,31.75)
hsCRP (mg/L) [median, (Q1, Q3)]	10.00 (5.32, 18.72)
CK (U/L) [median, (Q1, Q3)]	43 (30, 91)
CKMB-mass(μg/L) [median, (Q1, Q3)]	0.500 (0.075, 1.025)
cTnI(μg/L) [median, (Q1, Q3)]	0.000 (0.000, 0.025)
NT-proBNP (pg/ml) [median, (Q1, Q3)]	482.00 (73.25, 1,882.75)
Echocardiographic abnormality (*n*, %)	34 (15.1%)
Complications	
Hypertension (*n*, %)	51 (22.7%)
Diabetes (*n*, %)	32 (14.2%)
Coronary atherosclerosis (*n*, %)	17 (7.6%)
Chronic infection diseases^b^ (*n*, %)	36 (16.0%)
Infections during hospitalization (*n*, %)	85 (37.8%)

BMI, body mass index; PASI, psoriasis area and severity index; WBC, white blood cell; NEUT, neutrophil; Hbg, hemoglobin; Alb, albumin; LDH, lactic dehydrogenase; eGFR, estimated glomerular filtration rate; TC, total cholesterol; TG, triglyceride; HDL-C, high density lipoprotein cholesterol; LDL-C, low density lipoprotein cholesterol; ESR, erythrocyte sedimentation rate; hsCRP, high-sensitivity C-reactive protein; NT-proBNP, N-terminal pro-B-type natriuretic peptide.

^a^ Severity evaluation of EP is according to the previous work the proposed by our team. A moderate-to-severe EP patient exhibits at least two of the three following characteristics while a mild EP patient exhibits less than two characteristics: (i) body temperature higher than 37.3°C upon admission; (ii) swelling and exudation of more than half of the skin lesion or lower extremity oedema; (iii) superficial lymphadenopathy.

^b^ Chronic infections include HBV, tuberculosis, and Syphilis.

Regarding the symptoms of the participants related to cardiovascular disease, 129 had bilateral, lower-extremity concave edema, 10 had palpitations, 9 had chest tightness and showed breath-holding, 3 had oliguria, and 2 had precordial discomfort. Serum CK activity was measured in 33 participants, who had a median activity of 43 (30, 91) U/L; CKMB-mass was measured in 30 participants, who had a median activity of 0.500 (0.075, 1.025) μg/L; cTnI was measured in 30 participants, who had a median concentration of 0.000 (0.000, 0.025) μg/L; and NT-proBNP was measured in 30 participants, who had a median concentration of 482.00 (73.25, 1,882.75) pg/ml. Thirty-four patients had electrocardiographic abnormalities, of which sinus tachycardia was the most common.

### Prevalence of heart failure and associated factors

Twenty-five participants with EP were diagnosed with heart failure, accounting for 11.1% of all the participants, and they had a mean age of 58.2 years. None of the 25 patients had a history of heart failure before hospitalization. Therefore, the patients were diagnosed with EP earlier than heart failure. Among the 25 EP patients with heart failure, 17 patients were diagnosed by internal consultant; 8 patients were diagnosed by dermatologists without internal consultation. The participants were allocated to two groups according to whether they had heart failure or not, then the sex, age, morbidity, clinical manifestations, laboratory test data, and prevalence of comorbidities were compared between the groups (see Tables 2, 3). We found that patients with EP and heart failure were significantly older than those without heart failure (58.2 years vs. 46.2 years, *p *= 0.001). There was a significantly higher prevalence of a history of coronary heart disease in participants with heart failure than in those without (32.0% vs. 4.5%, *p *< 0.001). With respect to the clinical manifestations, the prevalence of fever (48.0% vs. 23.0%, *p *= 0.007) and bilateral lower limb edema (76.0% vs. 55.0%, *p *= 0.045) was significantly higher in participants with heart failure than in those without. The prevalence of moderate-to-severe EP was also significantly higher in participants with heart failure than in those without (64.0% vs. 33.5%, *p *= 0.003).

**Table 2:** Comparison of clinical characteristics between patients with heart failure or without.

	Heart failure (+)	Heart failure (−)	*p*-value	Odds ratio	95% CI
	*n* = 25	*n* = 200			
Male (*n*, %)	15 (60.0%)	149 (74.5%)	0.124	0.513	0.217–1.215
Age (years)	58.2 ± 14.8	46.2 ± 16.5	0.001**	/	/
Smoke history (*n*, %)	15 (60.0%)	95 (47.5%)	0.238	1.658	0.711–3.867
Alcohol use (*n*, %)	4 (16.0%)	40 (20.0%)	0.634	0.762	0.248–2.344
Length of stay (days) (average ± SD)	27.1 ± 19.3	25.2 ± 13.9	0.535	/	/
Age of psoriasis onset (years) (average ± SD)	40.4 ± 17.5	32.1 ± 16.5	0.020*	/	/
Duration of previous psoriasis (years) (median, qualities)	17.7 ± 13.3	14.0 ± 11.1	0.133	/	/
EP duration (Weeks)	4 (2, 8)	4 (3, 12)	0.112	/	/
Fever (*n*, %)	12 (48.0%)	46 (23.0%)	0.007**	3.090	1.320–7.237
Lower limbs edema (*n*, %)	19 (76.0%)	110 (55.0%)	0.045*	2.591	0.993–6.761
Lymph node enlargement (*n*, %)	8 (32.0%)	64 (32.0%)	1.000	1.000	0.410–2.438
Moderate to severe EP (*n*, %)	16 (64.0%)	67 (33.5%)	0.003**	3.529	1.482–8.405
Obesity (*n*, %)	3 (12.0%)	18 (9.0%)	0.627	1.379	0.376–5.058
Diabetes (*n*, %)	3 (12.0%)	29 (14.5%)	0.736	0.804	0.226–2.860
Hyperlipidemia (*n*, %)	10 (40.0%)	40 (20.0%)	0.023*	2.667	1.115–6.377
Hypertension (*n*, %)	8 (32.0%)	43 (21.5%)	0.237	1.718	0.695–4.249
Infection (*n*, %)	14 (56.0%)	71 (35.5%)	0.046*	2.312	0.997–5.362
Coronary atherosclerosis (*n*, %)	8 (32.0%)	9 (4.5%)	<0.001***	9.987	3.413–29.227

OR, odds ratio.

* *p *< 0.05, **p < 0.01, ***p < 0.001.

**Table 3:** Comparison of laboratory characteristics between patients with heart failure or without.

	Heart failure (+)	Heart failure (−)	*p*-value	Odds ratio	95% CI
	*n* = 25	*n* = 200			
WBC count (×10^9^/L)	9.54 ± 3.13	8.34 ± 3.15	0.075	/	/
WBC elevation (*n*, %)	12 (48.0%)	58 (29.0%)	0.053	2.260	0.974–5.245
NEUT count (×10^9^/L)	6.84 ± 3.21	5.52 ± 2.72	0.026*	/	/
NEUT elevation (*n*, %)	10 (40.0%)	35 (17.5%)	0.008**	3.143	1.304–7.572
Hgb (g/L)	116.4 ± 23.8	127.5 ± 19.1	0.009**	/	/
Anemia (*n*, %)	15 (60.0%)	44 (22.0%)	<0.001***	5.318	2.234–12.660
Alb (g/L)	31.7 ± 5.9	35.2 ± 5.5	0.004**	/	/
Hypoalbuminemia (*n*, %)	16 (64.0%)	84 (42.0%)	0.037*	2.455	1.035–5.822
LDH (U/L)	231.2 ± 85.7	201.4 ± 76.1	0.106	/	/
ESR (mm/h)	25 (12, 55)	16 (8, 28)	0.052	/	/
hsCRP (mg/L)	43.2 (6.8, 106.4)	8.2 (3.8, 20.5)	0.005**	/	/
Hyperlipidemia (*n*, %)	10 (40.0%)	40 (20.0%)	0.023*	2.667	1.115–6.377

Elevated peripheral white blood cell (WBC) count was defined as absolute count of WBC >9.5 × 109/L. Elevated peripheral neutrophil (NEUT) count was defined as absolute count of NEUT >7.5 × 109/L. Anemia was defined as hemoglobin <120 g/L in men and <110 g/L in women. Hypoalbuminemia was defined as serum albumin <35 g/L.

OR, odds ratio; WBC, white blood cell; NEUT, neutrophi; Hbg, hemoglobin; Alb, albumin; Glu, glucose; LDH, lactic dehydrogenase; ESR, erythrocyte sedimentation rate; hsCRP, high-sensitivity C-reactive protein.

* *p *< 0.05, **p < 0.01, ***p < 0.001.

In respect of laboratory test data, the peripheral neutrophil count was significantly higher in participants with heart failure than in those without (6.84 × 10^9^/L vs. 5.52 × 10^9^/L, *p *= 0.026). The prevalence of anemia was significantly higher in participants with EP and heart failure (60.0% vs. 22.0%, *p *< 0.001) than in those without heart failure, as was the prevalence of hypoalbuminemia (64.0% vs. 42.0%, *p *= 0.037). The participants with heart failure also had significantly higher concentrations of hsCRP than those without (43.2 mg/L vs. 8.2 mg/L, *p *= 0.005). Finally, the prevalence of hyperlipidemia (40.0% vs. 20.0%, *p *= 0.023) and that of infection during hospitalization were significantly higher in participants with heart failure than in those without (56.0% vs. 35.5%, *p *= 0.046).

The final logistic regression model obtained was statistically significant (*χ*^2^ = 29.323, *p* < 0.001). This model was able to correctly classify 90.2% of the study subjects. The sensitivity of the model was 99.0%, specificity was 20.0%, positive predictive value was 71.4%, and negative predictive value was 95.2%. A total of three independent variables included in the model were statistically significant. The risk of heart failure increased with age, with statistical significance (OR = 1.039, 95% CI: 1.007–1.072, *p* = 0.018). Besides, the risk of heart failure significantly increased in patients with anemia compared with those without anemia (OR = 4.157, 95% CI: 1.660–10.410, *p* = 0.002). Thirdly, history of coronary heart disease also significantly increased the risk of heart failure (OR = 4.582, 95% CI: 1.419–14.796, *p* = 0.011).

### Serum concentration of FGF23

**Figure 1:**
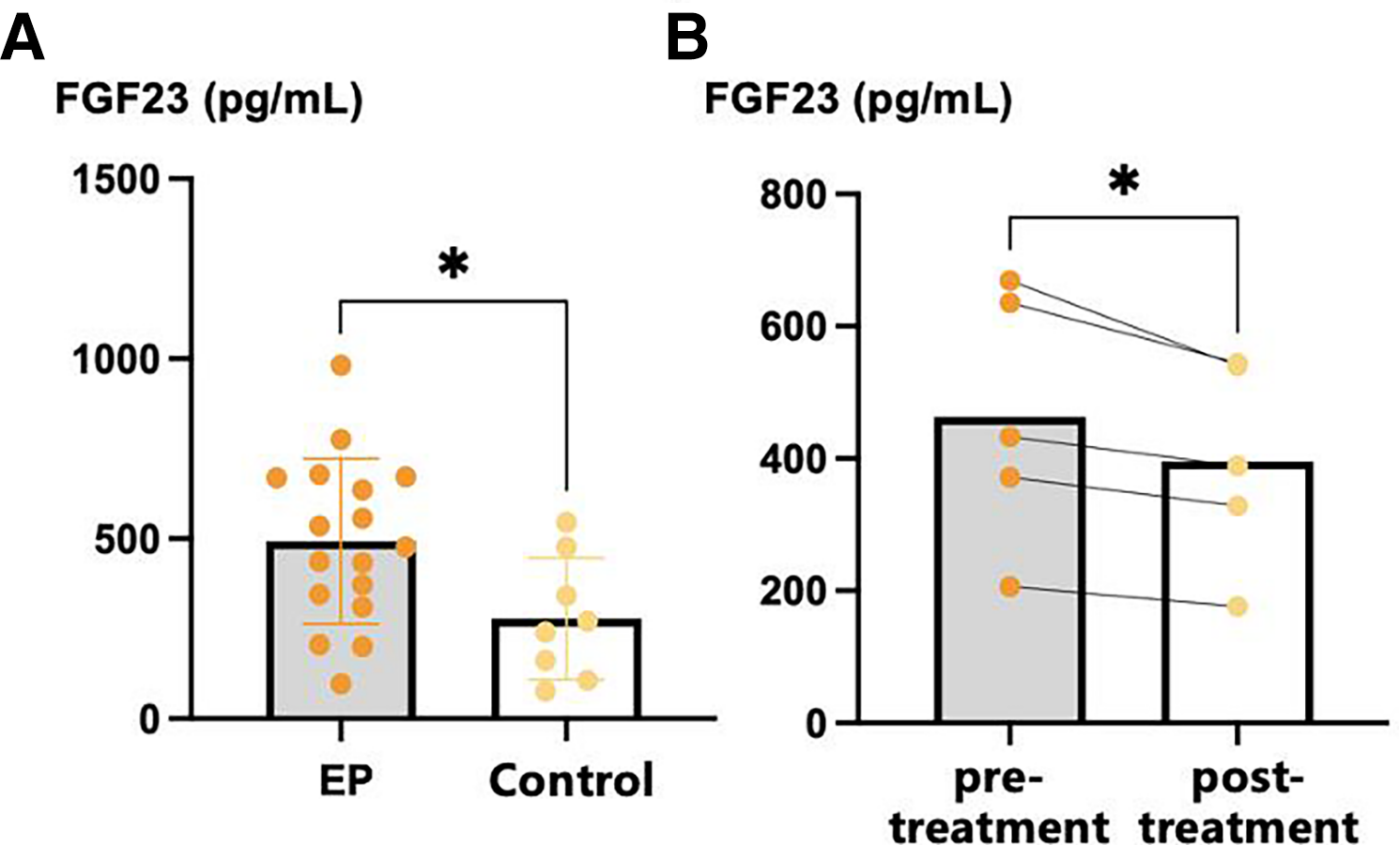
Serum FGF23 levels of EP patients and healthy controls. (**A**) Serum FGF23 levels of EP patients and healthy controls; (**B**) pre-treatment and post-treatment FGF23 levels of EP patients. *p < 0.05, **p < 0.01, ***p < 0.001. FGF23, fibroblast growth factor 23.

The mean serum FGF23 concentration of participants with EP was significantly higher than that of healthy controls (493.1 pg/ml vs. 277.8 pg/ml, *p *= 0.027). The therapies provided to the five participants with EP for whom post-treatment serum samples were collected were as follows: acitretin 30 mg qd (*n* = 3), acitretin 20 mg qd combined with methotrexate 5 mg qw (*n* = 1), and cyclosporine 50 mg tid (*n* = 1). The serum FGF23 concentration was significantly lower following these treatments (395.7 pg/ml vs. 463.1 pg/ml, *p *= 0.022) in these participants (Figure 1).

**Figure 2:**
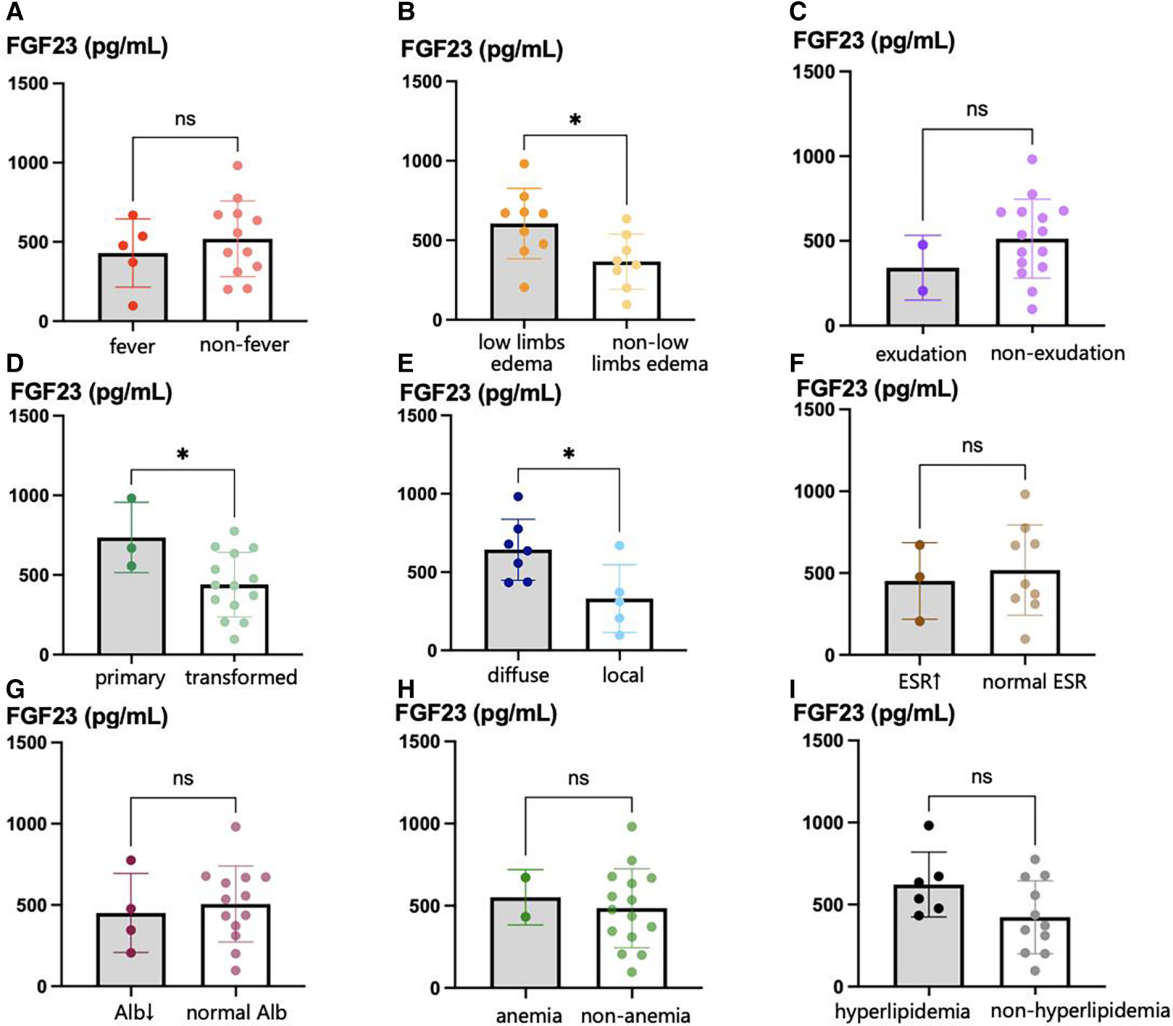
Serum FGF23 levels in EP patients with different clinical characteristics. (**A**) serum FGF23 levels in EP patients with fever and those without fever; (**B**) serum FGF23 levels in EP patients with lower limbs edema and those without lower limbs edema; (**C**) serum FGF23 levels in primary EP patients with exudation and those without exudation; (**D**) serum FGF23 levels in primary EP patients and those transformed from other types of psoriasis; (**E**) serum FGF23 levels in EP patients with diffuse initial skin lesions and those with local diffuse skin lesions; (**F**) serum FGF23 levels in EP patients with elevated ESR and those with normal ESR; (**G**) shows serum FGF23 levels in EP patients with hypoalbuminemia and those with normal serum albumin level; (**H**) serum FGF23 levels in EP patients with and without anemia; (**I**) serum FGF23 levels in EP patients with hyperlipidemia and those without hyperlipidemia. *p < 0.05, **p < 0.01, ***p < 0.001.

A comparison of the serum FGF23 concentrations of the participants with EP and differing clinical characteristics is shown in Figure 2. The serum FGF23 concentration of participants with primary EP was significantly higher than that of participants with EP that had developed from other types of psoriasis (736.1 pg/ml vs. 441.0 pg/ml, *p *= 0.039). Regarding the initial site of the skin lesions, the FGF23 concentration of participants with EP who had skin lesions covering their bodies was significantly higher than in participants who initially had localized lesions (642.6 pg/ml vs. 330.7 pg/ml, *p *= 0.026). The relationships between the FGF23 concentration and other parameters, including the demographic and clinical characteristics and laboratory findings, are shown in Table 4. We found that the serum FGF23 concentrations of participants with EP negatively correlated with the duration of EP (correlation coefficient −0.578, *p *= 0.015) and positively correlated with the degree of edema (correlation coefficient 0.714, *p *= 0.001).

**Table 4:** Correlation analysis between blood FGF23 levels and clinical characteristics.

Characteristics		*r*	*p*-value	Characteristics		*r*	*p*-value
Demographic characteristics	Age (years)	0.106	0.686	Laboratory examinations	WBC (×10^9^/L)	−0.055	0.835
	Male	−0.283	0.271		NEUT (×10^9^/L)	−0.004	0.989
	BMI (kg/m^2^)	0.121	0.756		Hbg (g/L)	−0.068	0.797
	Smoke history	0.075	0.774		Anemia	0.112	0.669
	Alcohol use	0.037	0.887		PLT (×10^9^/L)	−0.239	0.355
	Duration of psoriasis (years)	−0.273	0.289		Alb (g/L)	−0.109	0.689
Clinical characteristics	Primary EP	0.283	0.271		Hypoalbuminemia	−0.085	0.746
	Site of primary lesion	0.661	0.019		Creatine (μmol/L)	−0.059	0.822
	EP duration (Weeks)	−0.578	0.015*		eGFR (ml/min/1.73 m^2^)	−0.064	0.807
	Exudation	−0.224	0.388		Renal dysfunction	0.204	0.432
	Palmoplantar keratosis	0.410	0.102		Glucose (mmol/L)	−0.244	0.346
	Fever	−0.158	0.544		LDH (U/L)	−0.120	0.778
	Lower limbs edema	0.577	0.015		ESR (mm/h)	0.043	0.893
	Degrees of lower limb edema	0.714	0.001**		ESR elevation	−0.084	0.796
	Severity score	0.142	0.587		hsCRP (mg/L)	0.344	0.330
	Heart rate (beats per minute)	0.424	0.402		hsCRP elevation	0.284	0.426
	Hyperlipidemia	0.377	0.136				

BMI, body mass index; WBC, white blood cell; NEUT, neutrophil; Hgb, hemoglobin; Alb, albumin; AST, aspartate transaminase; ALT, alanine aminotransferase; LDH, lactic dehydrogenase; Cr, creatinine; eGFR, estimated glomerular filtration rate; Glu, glucose; ESR, erythrocyte sedimentation rate; hsCRP, high-sensitivity C-reactive protein; IL-6, interleukin-6; IL-8, interleukin-8; IL-17A, interleukin-17A; TNF-α, tumor necrosis factor alpha; *r*, correlation coefficient; OR, odds ratio.

* *p *< 0.05, **p < 0.01.

## Discussion

EP is a rare, severe subtype of psoriasis, characterized by erythematous, edematous, often exfoliative lesions that affect over 90% of body surface area (5). EP is associated with a substantial risk of morbidity due to transepidermal fluid, nutrient loss, secondary infections, multi-organ failure and even death in severe cases (26). The National Psoriasis Foundation established guidelines for EP management in 2010 (6), but is limited by a paucity of high-quality evidence due to the rarity and potentially emergent nature of EP (27). The management of EP is still challenging and mainly based on personal experience and literature reports. Biologic therapies may have a growing role in the management of EP, showing a generally safe profile, linked to rapid and effective action on EP manifestations. However, their role should be better evaluated in further and larger clinical trials and real-life studies (2, 28–30). Despite the fact that there are various treatment options for EP, the consensus has advised that the first step should be evaluations and corrections of fluid, electrolyte, protein imbalances, secondary infections, and vital organ damage (27).

In the present study, we retrospectively analyzed data relating to 225 patients with EP who were admitted to Peking Union Medical College Hospital between 2005 and 2021. We found that 11.1% of these patients had heart failure. Several previous studies have shown that patients with EP can develop high-output heart failure (13, 14), and that diffuse cutaneous inflammation can cause vasodilation and an increase in vascular permeability, leading to a reduction in blood pressure and peripheral edema (13). If patients have structural or functional heart defects that prohibit an increase in cardiac output, the persistent hypotension will lead to severe compensatory fluid retention, which might be the underlying cause of the pulmonary edema and congestive heart failure (31). A Danish population study by Khalid et al. (32) showed that patients with psoriasis are at significantly higher risk of heart failure than the general population. In addition, the level of risk was found to be associated with the severity of the disease because patients with mild psoriasis and severe psoriasis had adjusted hazard ratios (HRs) of 1.22 (95% CI: 1.16–1.29) and 1.53 (95% CI: 1.34–1.74), respectively. Heart failure is the leading cause of death in patients with cardiovascular disease, and a previous study showed that approximately 50% of patients with heart failure die within 5 years of discharge and have a prognosis comparable to that of many malignancies (33). Therefore, clinicians should be aware of the risk of heart failure in patients with severe psoriasis, and especially in those with EP.

In the present study, we found that the prevalence of hyperlipidemia was significantly higher in patients with EP and heart failure than in patients with EP but no heart failure, but there were no significant differences in the prevalence of conventional cardiovascular disease risk factors (hypertension, diabetes, and obesity) between the two groups. This suggests that the high risk of heart failure in patients with EP cannot be fully explained by the coexistence of conventional cardiovascular risk factors in patients with EP. Consistent with this, Gelfand et al. (34) found by means of a large cohort study that psoriasis is associated with a higher risk of myocardial infarction after adjustment for major cardiovascular risk factors (BMI, smoking, hypertension, diabetes, and hyperlipidemia).

We also found that patients with EP and heart failure have a higher prevalence of fever and high concentrations of inflammatory biomarkers, suggesting that this group of patients with EP may have more serious systemic inflammation. Consistent with this, the prevalence of heart failure in participants with moderate-to-severe EP was significantly higher than that in participants with mild EP, suggesting that the risk of heart failure in participants with EP is related to disease severity. Several previous studies have shown that the risk of myocardial infarction is positively related to the severity of psoriasis (34–36).

Notably, we found a higher prevalence of infection in participants with EP and heart failure, which may explain the significantly higher peripheral blood neutrophil count, concentrations of inflammatory cytokines, and prevalence of fever in these patients. Infection is a common cause of a worsening of heart failure and can lead to acute decompensation in severe cases (37). In the present study, 37.8% of patients with EP were found to have infections during hospitalization, suggesting that it is a common complication in patients with EP, and therefore infections should be monitored for and properly managed to avoid the development or worsening of heart failure.

We also found that the prevalence of anemia in participants with EP and heart failure was significantly higher than that in participants with EP but no heart failure. Previous studies have shown that 27% of patients with psoriasis have anemia, mainly because of folic acid and iron deficiency (38). Patients with severe anemia may show compensatory increases in circulatory volume and cardiac load, owing to lower oxygen-carrying capacity of the blood. In addition, it has been found that anemia is an independent risk factor for hospitalization and mortality in patients with heart failure (39). Therefore, patients with EP and both heart failure and anemia should be managed carefully to avoid a poor prognosis.

FGF23 was initially found to be a regulator of phosphate and vitamin D metabolism, and was also shown to be associated with various genetic disorders and chronic kidney disease (40, 41). Recent studies have shown that FGF23 not only is associated with the development of cardiac complications in patients with CKD but also may be independently associated with cardiac disease (41). Large epidemiological studies have shown a strong associations between FGF23 concentration and the risks of cardiovascular disease, especially heart failure and stroke, and mortality (42, 43). Furthermore, these associations were shown to persist after adjustment for renal dysfunction in another study (44). The mechanism explaining the relationship between FGF23 and the development of cardiac disease remains unclear. However, previous studies have shown that high FGF23 concentration may be associated with endothelial dysfunction and activation of the renin-angiotensin-aldosterone system (RAAS) (16, 45). In addition, Silswal et al. (46) found that FGF23 induces endothelial dysfunction by increasing superoxide and reducing nitric oxide bioavailability in mouse models, thereby directly inhibiting vasodilation and inducing endothelial injury.

In the present study, we found that the serum FGF23 concentrations of patients with EP are significantly higher than those of healthy individuals matched for age, sex, and a history of hypertension, diabetes, or hyperlipidemia, suggesting that patients with EP may be at higher risk of heart failure than the healthy population. Few studies have investigated the serum FGF23 concentrations of patients with psoriasis, but Gökhan et al. (21) found a mean serum FGF23 concentration of 272.8 pg/ml in patients with psoriasis vulgaris, which was significantly higher than that in healthy controls. However, they found no significant association between FGF23 concentration and PASI score. In addition, the serum FGF23 concentration significantly decreased during the treatment of patients with EP, suggesting that the serum FGF23 concentration may be related to the severity of psoriasis. Therefore, we speculate that it may be possible to reduce the risk of heart failure in patients with EP by controlling their skin lesions.

Only one of the 17 patients with EP in the present study had an eGFR slightly lower than 90 ml/min/1.73 m^2^, and there was no significant relationship between FGF23 concentration and eGFR. These findings strongly suggest that the high FGF23 concentrations in patients with EP identified in the present study were not secondary to renal insufficiency. In addition, higher serum FGF23 concentrations were found in patients with an acute course of EP and generalized skin lesions, which may be explained by a rapid increase in cardiac load secondary to the sudden appearance of large skin lesions in these patients. In addition, we found a positive correlation between FGF23 concentration and the severity of bilateral lower limb edema. Therefore, the findings of the present study suggest that patients with acute EP, those who rapidly develop generalized lesions, and those with severe bilateral lower extremity edema are at higher risk of heart failure and should be carefully monitored.

The present study had some limitations. First, it was a single-center, retrospective study. In addition, most of the participants without heart failure-related symptoms did not undergo further cardiac investigations. Therefore, there is a possibility that patients with subclinical or mild heart failure were not diagnosed. Finally, we only included patients diagnosed with heart failure during hospitalization. Furthermore, the sample size in FGF23 test was relatively small, and the decrease of FGF23 did not adequately indicate a decrease in heart failure risk. Therefore, prospective studies in which cardiac examinations are routinely performed on newly admitted patients with EP should be conducted in the future.

In conclusion, we found that patients with EP are at risk of heart failure, and advanced age, anemia, hypoalbuminemia, concurrent infection, and a previous history of coronary artery disease may represent risk factors. In addition, we found that patients with EP had significantly higher serum concentrations of FGF23, a marker of incident heart failure, than healthy individuals. Our findings imply that physicians should be aware of the risk of heart failure in patients with EP, and that serum FGF23 may represent a marker of the risk of heart failure in such patients. However, larger, prospective controlled studies with extended follow-up periods are needed to confirm these findings.

## Data Availability

The raw data supporting the conclusions of this article will be made available by the authors, without undue reservation.
